# Validating an Automated Nucleic Acid Extraction Device for *Omics in Space* Using Whole Cell Microbial Reference Standards

**DOI:** 10.3389/fmicb.2020.01909

**Published:** 2020-08-21

**Authors:** Camilla Urbaniak, Season Wong, Scott Tighe, Arunkumar Arumugam, Bo Liu, Ceth W. Parker, Jason M. Wood, Nitin K. Singh, Dana J. Skorupa, Brent M. Peyton, Ryan Jenson, Fathi Karouia, Julie Dragon, Kasthuri Venkateswaran

**Affiliations:** ^1^NASA Jet Propulsion Laboratory, California Institute of Technology, Pasadena, CA, United States; ^2^AI Biosciences, College Station, TX, United States; ^3^University of Vermont, Burlington, VT, United States; ^4^Montana State University, Bozeman, MT, United States; ^5^IRPI LCC, Portland, OR, United States; ^6^NASA Ames Research Center, Moffett Field, CA, United States

**Keywords:** International Space Station (ISS), microbial monitoring, automated DNA extraction, microgravity (μg), extreme environments

## Abstract

NASA has made great strides in the past five years to develop a suite of instruments for the International Space Station in order to perform molecular biology in space. However, a key piece of equipment that has been lacking is an instrument that can extract nucleic acids from an array of complex human and environmental samples. The *Omics in Space* team has developed the μTitan (simulated micro(**μ**) gravity **t**ested **i**ns**t**rument for **a**utomated **n**ucleic acid) system capable of automated, streamlined, nucleic acid extraction that is adapted for use under microgravity. The μTitan system was validated using a whole cell microbial reference (WCMR) standard comprised of a suspension of nine bacterial strains, titrated to concentrations that would challenge the performance of the instrument, as well as to determine the detection limits for isolating DNA. Quantitative assessment of system performance was measured by comparing instrument input challenge dose vs recovery by Qubit spectrofluorometry, qPCR, Bioanalyzer, and Next Generation Sequencing. Overall, results indicate that the μTitan system performs equal to or greater than a similar commercially available, earth-based, automated nucleic acid extraction device. The μTitan system was also tested in Yellowstone National Park (YNP) with the WCMR, to mimic a remote setting, with limited resources. The performance of the device at YNP was comparable to that in a laboratory setting. Such a portable, field-deployable, nucleic extraction system will be valuable for environmental microbiology, as well as in health care diagnostics.

## Introduction

All terrestrial organisms exposed to the environmental conditions of space are subjected to reduced gravity, high-energy charged particles, high UV levels, low pressure, and large changes in temperature. When living in such an environment, humans can become immunocompromised (Mehta et al., 2017), microbial behavior can be changed (Kim et al., 2013), and host-microbial interactions can be altered (Foster et al., 2014). To diagnose patients or to perform microbial monitoring on Earth, samples are immediately sent to a laboratory for testing and results can be obtained in a day or two. However, samples collected from the International Space Station (ISS) will take from 2 to 4 months to return back to Earth before analyses can be performed (Pierson et al., 2012), and it would not be practical for future long-term space missions to send samples back to Earth for analysis. To make exploring and living in space feasible, instruments that automate laboratory analysis will need to be developed. Since most molecular diagnostic methods for assessing and monitoring health are based on nucleic acids (Dwivedi et al., 2017), focused efforts to design portable nucleic acid-based molecular diagnostic assays (Chan et al., 2016a, c; Priye et al., 2016), compatible with microgravity, are a high priority.

In 2016, Dr. Kathleen Rubins, a NASA astronaut, became the first person to sequence DNA on the ISS, using the Oxford Nanopore MinION sequencer (Castro-Wallace et al., 2017). While this was a great milestone for space molecular biology, the DNA that was sequenced had already been extracted and the metagenome libraries prepared on Earth before sending to space for sequencing (Castro-Wallace et al., 2017). In another flight project, bacterial DNA, which also had been isolated on Earth, was sent to the ISS and successfully amplified with the miniPCR^TM^ (Boguraev et al., 2017). More recently, methods have been developed to allow for DNA and RNA extraction to occur on the ISS before *in situ* downstream analyses. For example, with the Genes in Space-3 project, cultured bacterial cells that had been isolated from around the ISS were lysed in a thermocycler and the DNA amplified before being sequenced on the MinION (NASA, 2017). With the WetLab-2 research platform, RNA was successfully isolated from *Escherichia coli* cultures by lysing the cells with bead beating and then capturing the released RNA with the RNAexpress^TM^ column, after which the isolated RNA was analyzed with qPCR (Parra et al., 2017). The current methods are a tremendous advancement for space genomics because they allow for a complete sample-to-analysis process aboard the ISS, but they also have drawbacks. These techniques are manual and take up crew time, are not high throughput, and are better suited for pure cultures rather than complex, mixed microbial communities, as they are not efficient for low biomass samples and the nucleic acids are not pure due to the leftover cell debris. To address these limitations, the “Omics in Space” team developed μTitan (simulated micro(**μ**) gravity **t**ested **i**ns**t**rument for **a**utomated **n**ucleic acid extraction), an automated sample processing system, that is microgravity compatible, and can process multiple complex samples simultaneously in an easy to use streamlined manner. Although there are automated sample processing devices commercially available for use on the ground to process multiple samples simultaneously (e.g., Maxwell^TM^ 16; Promega, Madison, MI), these instruments are not microgravity compatible nor are they lightweight and compact.

The objectives of this study were two-fold; (i) to develop an automated nucleic acid extraction device that would be microgravity compatible and thus could be used on the ISS, and (ii) to validate system performance of this device (i.e., μTitan) using a whole cell microbial reference (WCMR) standard and to compare this performance against the commercially available, widely used, automated nucleic extraction system, Maxwell^TM^. The sensitivity and selectivity of the μTitan system and how it compared to the Maxwell^TM^ system was assessed by Qubit (for DNA yield), qPCR (for 16S rRNA gene copy numbers) and the Bioanalyzer (for extracted DNA length). In addition, shotgun metagenomics sequencing was performed on two platforms, Illumina MiSeq and Oxford Nanopore MinION in order to assess the ability to produce reliable sequencing data, in terms of diversity and relative proportions of the bacteria present in the WCMR.

## Materials and Methods

### Preparing the Whole Cell Microbial Reference Standard

The whole cell microbial reference (WCMR) standard used to validate the μTitan system consists of nine bacteria, five of which are Gram positive and four of which are Gram negative. The strains used, their amounts, and other metadata are listed in Table 1. The WCMR was produced as part of the Association of Biomolecular Resource Facilities (ABRF) metagenomics research group (Tighe et al., 2017). Briefly, individual bacteria were cultured on either tryptic soy agar or marine 2216 agar until early log phase growth was achieved, followed by harvesting and resuspension in 1x phosphate buffered saline (PBS) without calcium or magnesium. The cell suspension was washed twice with 1x PBS followed by vortexing, to ensure that cells were in a unicellular suspension and not aggregated (confirmed by microscopy) before the fixation step with ethanol. Fixation was performed by adding 100% ethanol in a drop wise fashion while vortexing until the final concentration was between 90–95%. Cells were allowed to settle overnight, and the supernatant was discarded to remove any free-floating DNA. The final sample was resuspended in fresh 90% ethanol and stored at 4°C.

**TABLE 1:** Metadata of the nine bacteria present in the whole cell microbial reference standard used for validation.

Organism	ATCC Number	Gram stain	Genome size (Mb)	Reference	Volume Combined	Cells/μl	SD
*Staphylococcus epidermidis* PCI 1200	12228	+	3.5	GCF_000007645.1_ASM764v1	200 μl	1.2 × 10^6^	1.8 × 10^5^
*Pseudomonas fluorescens* F113	13525	−	8.7	GCF_000237065.1_ASM23706v1	150 μl	7.9 × 10^5^	1.1 × 10^5^
*Escherichia coli* K-12 substr. MG1655	700926	−	5.6	GCF_000005845.2_ASM584v2	250 μl	9.9 × 10^5^	1.4 × 10^5^
*Chromobacter violaceum* NCTC 9757	12472	−	2.7	GCF_000007705.1_ASM770v1	100 μl	2.3 × 10^6^	3.2 × 10^5^
*Micrococcus luteus* NCTC 2665	4698	+	3.2	GCF_000023205.1_ASM2320v1	50 μl	5.6 × 10^5^	9.5 × 10^4^
*Pseudoalteromonas haloplanktis* TAC125	35231	−	4.0	GCF_000238355.1_Phal_1.0	300 μl	8.9 × 10^4^	5.3 × 10^3^
*Bacillus subitilis* subsp. subtilis str. 168	23857	+	5.8	GCF_000009045.1_ASM904v1	250 μl	8.3 × 10^5^	1.7 × 10^5^
*Halobacillus halophilus* DSM 2266	3567	+	5.6	GCF_000284515.1_ASM28451v1	250 μl	4.0 × 10^5^	5.7 × 10^4^
*Entercoccus faecalis* OG1RF	47077	+	3.6	GCF_000007785.1_ASM778v1	200 μl	8.9 × 10^4^	3.8 × 10^4^

Enumeration of each individual ethanol fixed bacterial suspension was performed by diluting 1 μl of the suspension in 48 μl of 1X PBS and 1 μl of 0.1% sodium dodecyl sulfate. Staining was performed by adding 1 μl of Sytox staining reagent, 1 μl of SYBR green (2.5X), 1 μl of enhancer, and 7 μl of buffer (all reagents from Logos BioSystems, Seoul, S. Korea) to this dilution and incubating in the dark at room temperature for 5 min. Enumeration was performed by adding 4 μl of the stained cell preparation to a INCYTO counting slide (INCYTO C-Chip Hemocytometers, DHC-S025) and counted at 400x magnification using 485 nm/525 nm epifluorescent microscopy according to the manufacturer’s protocol. Combining the individual bacterial preparation into one pool was based on a staggered design. Cell concentrations ranged from 10^4^ to 10^6^ cells/μl, depending on the organism (Table 1), resulting in a mixed microbial community containing 7.3 × 10^6^ total cells/μl.

Before using the WCMR to validate the μTitan system, a controlled laboratory extraction of DNA was performed using a modified Qiagen procedure by first washing the cells in 1× PBS followed by enzymatic digestion of the cell walls with 20 μl of Metapolyzyme (MPZ) [stock concentration = 10μg/μl] (Millipore Sigma, St. Louis, MO, United States) at 35°C for 4 h, after which DNA extraction was completed using a QIAamp DNA Micro Kit (Qiagen 56304, Germantown, MD) with a pretreatment bead beating step (Matrix A beads MP biomedical 116910050CF) to increase lysis efficiency. Measurements from a Qubit spectrofluorometer indicated that 3.16 ng of DNA was obtained per 10^6^ cells.

### Overview of μTitan

**FIGURE 1:**
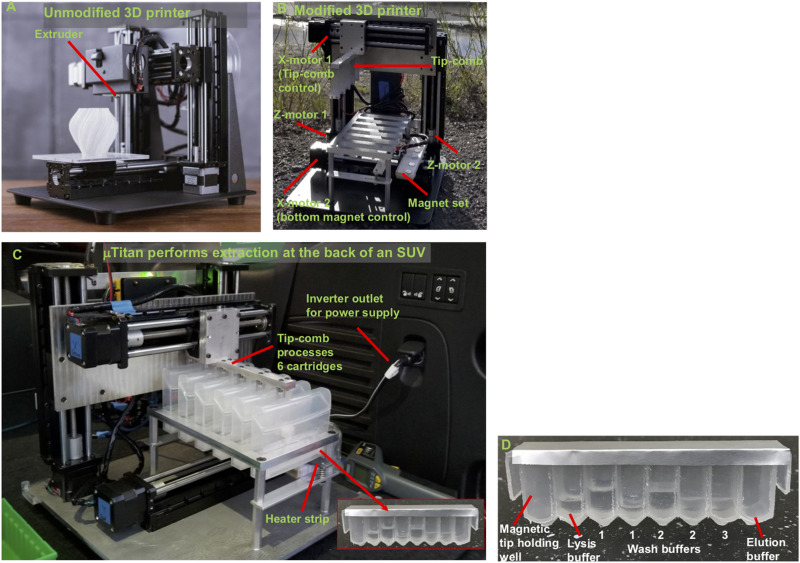
Concept of converting a low-cost 3D printer to perform rapid and automated NA isolation. **(A)** A typical FMD type 3D printer. **(B)** A 3D printer turned extraction device. The extruder of a 3D printer was removed to allow the adaptors to be mounted. The magnetic particle processing tip-comb (tip-comb) was attached on the mount. Its vertical and lateral movements are controlled by two Z-motors and one upper X-motor controls. Another X-motor control below the extraction cartridges enable resuspension of magnetic particles for NA binding and washing (see description later). The tip-comb has 6 fingers with each finger has magnets to use magnetic coupling to externally control the extraction tips inside an extraction cartridge to perform extraction. There is no direct contact between the magnets, the samples, and MPs. Six samples can be processed simultaneously under the same protocol program. The heated strip under the elution wells uses the 3D printer’s extruder heater and thermistor to provide precise temperature control for heated NA elution. **(C)** μTitan placed inside the cargo area of an SUV. **(D)** A reagent strip that has been pre-filled with the appropriate extraction reagents.

The μTitan system is based on a fused deposition modeling 3D printer in which the motion and temperature controls have been repurposed for automated NA extraction, with the capability to perform both heated incubation and NA amplification (Figures 1A,B). The system is portable and robust and was able to perform extractions while being transported in the back of a sports utility vehicle (SUV) at highway speed (Figure 1C). The extraction efficiency of *Chlamydia trachomatis* DNA from urine samples using an early prototype was demonstrated to be comparable to the spin column method from Qiagen (Chan et al., 2016a). Extractions of RNA from the Zika virus in urine and saliva samples also proved to be successful (Chan et al., 2016b).

### Mechanics of μTitan Designed for Use on the ISS

The current version of μTitan is 34×34×36 cm when encased with a cover. It weighs about 8 kg including the power adaptor. A laptop is currently used to operate the device, but the protocol program can be saved on a secure digital (SD) card if needed. A comparison between the Maxwell^TM^ and the μTitan specifications are presented in Table 2.

**TABLE 2:** Specifications of the μTitan and Maxwell^TM^ systems evaluated in this study.

	Maxwell^TM^	μTitan
# samples run simultaneously	16	6
Dimensions (W × D × H)	33 × 44 × 33 cm	34 × 34 × 36 cm
Weight	18.9 kg	8.0 kg
Typical protocol duration	37–40 min	10–20 min
Enclosed cartridge	No	Yes
Heated elution	Yes	Yes
Programmable protocols	Yes	Yes
Power requirements	100–240VAC, 50–60Hz, 2.1A	Power adaptor: 100–240VAC,50–60Hz, 1.8A Output to 12V and 10A

**FIGURE 2:**
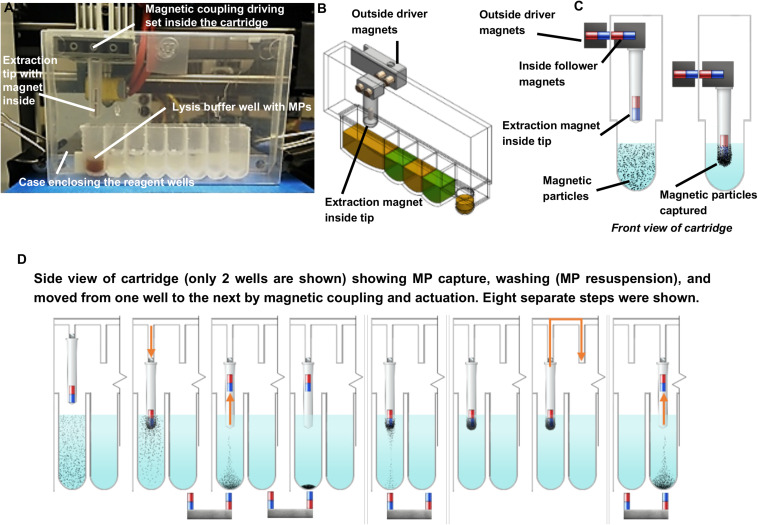
Extraction cartridge, magnetic coupling, and schematic of sample extraction. **(A–C)** External magnetic control and movement of sample by magnetic particles outside of extraction cartridge. **(D)** Detailed overview of magnetic handling, washing, and elution of NAs within the μTitan cartridge. The magnetic tip can move freely on the wall of the cartridge as actuated by the driver and follower magnets. The placement and the polarity of the magnets inside the extraction tip and those underneath the wells enable mixing and washing.

**FIGURE 3:**
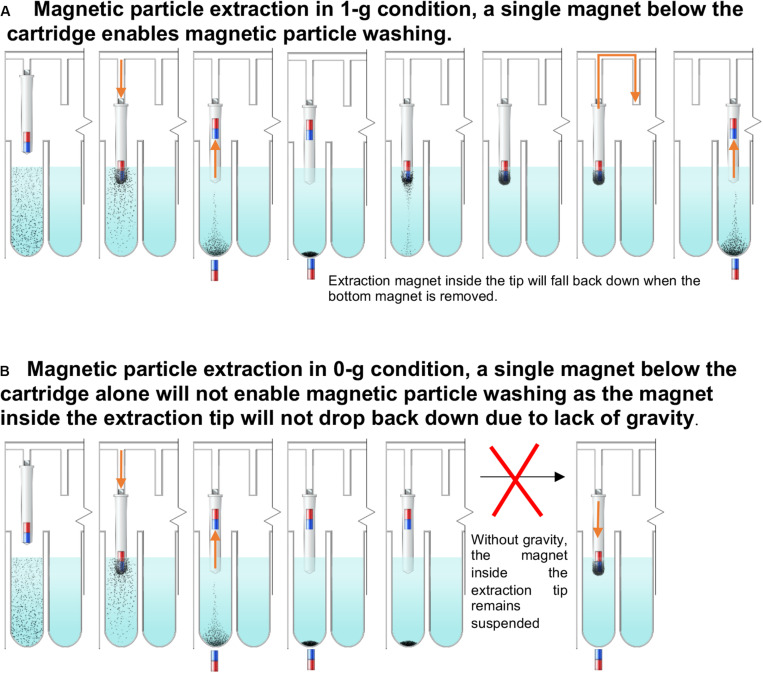
The μTitan cartridge at 1g vs reduced gravity. **(A)** Magnetic particle extraction in 1-g condition. Magnet inside of the extraction tip will fall back down to the bottom of the tip after the magnet with opposite polarity is removed. **(B)** In reduced gravity, the repelled extraction tip will remain afloat even after the bottom magnet is removed. Therefore, the paired-magnets mentioned in Figure SW3D were used.

Individually enclosed environments for sample extraction are typically used to prevent cross-contamination. This is essential in the ISS environment to avoid the unintentional release of extraction reagents and potentially harmful microbes. Currently, μTitan uses aluminum-foil-sealed, pre-filled reagent strips placed inside a strip holder that can be closed after sample input (Figure 1D). Using this approach, magnetic coupling can be used to perform extraction with multiple mounted cartridges. The operation of the coupling mechanism is similar to that of a magnetic fish tank cleaner, where the motion of NA extraction can be controlled inside the cartridge without direct contact from outside. Figure 2A shows the basic set-up where an 8-well reagent strip is placed inside an enclosure to become a cartridge. The mechanical movements inside each individual cartridge are enabled by a unique magnetic coupling approach (Figures 2B,C). These figures show how nucleic acid extraction is performed by the stepper motors that are programmed to automatically control the movement of the external magnets, which in turn drive the movement of the components inside the cartridge. The “driver magnets” outside of the cartridge precisely control the movement of “follower magnets” within the cartridge to process nucleic extraction and elution. The two sets of magnetic particles (MPs) are held together by the magnetic field that penetrates through the cartridge’s plastic wall. Synchronized motions of the magnetic tip-combs enable reproducible and controllable action inside each cartridge, including MP mixing. Figure 2D shows the process of effective mixing and washing of MPs. These external “driver magnets” allow for precise positioning of the “MP manipulating tip” located inside the cartridge. The bottom pair of magnets below the wells leads to release and capture of the MPs by this tip, allowing the MPs to move throughout the wash buffer, allowing for effective mixing and washing of these MPs. Under normal gravity, the magnet inside the tip falls back down when the bottom repelling magnet is removed (Figure 3A), but this is not the case in reduced gravity as the magnet inside the tip will just remain “floating” inside (Figure 3B). For the microgravity adapted machine, a set of opposing field magnets was installed to pull the extraction tip magnet down (Figure 2D). These innovative steps allow the elimination of pipetting in space under microgravity, which is difficult, laborious, and often results in mishandling.

### DNA Extraction Using μTitan and Maxwell^TM^

#### Pre-processing

One milliliter of the WCMR was pre-processed by incubating with 48 μl of 10 μg/μl MPZ for 15 min at 35°C, followed by 15 s of bead beating with Matrix Lysing E bead beating tubes (Mp Biomedicals) using a battery-powered oscillating power tool (Ryobi JobPLUS ONE 18V multi tool with P246 console & P570 Head attachment). The sample tube was placed and fixed tightly on the flat end of the blade using blue painters’ tape. In YNP, the WCMR was first subjected to bead beating (as above), then incubated with 25 μl MPZ for 1 h at 37°C, followed by another round of bead beating. Next, 100 μl of the pre-processed sample was added to either the Maxwell^TM^ or μTitan system. The high biomass samples (Tx_Max_High, Tx_μT_High, YNP_μT_High) correspond to an approximate input of 10^7^ cells, while the low biomass samples (Tx_Max_Low, Tx_μT_Low) correspond to an approximate input of 10^4^ cells. The “medium” biomass samples (YNP_μT_Med) corresponds to an approximate input of 10^6^ cells.

### μTitan Extraction

A commercially available magnetic particle–based NA isolation kit (NucliSENS Magnetic Particle Extraction Kit, bioMerieux, Durham, NC) was purchased, and the reagents from this kit were used to pre-fill the μTitan cartridges. These reagents included lysis buffer, magnetic particle solution, wash buffer #1, wash buffer #2, wash buffer #3, and elution buffer. The μTitan extraction protocol (e.g., volume, time of incubation, and number of repeated washing steps) is as follows: 100 μL of the pre-processed sample was added to the second well of a μTitan cartridge containing 400 μL of lysis buffer and incubated for 10 min. Eight microliters of NucliSENS magnetic particles, for capturing NAs, were then added to the lysed sample solution in the second well. The magnetic particles were intermittently mixed for 10 min, allowing for NA to bind to the MPs. The cartridge was then secured on the μTitan system. An extraction tip mounted on the follower magnet set was placed on the first well of the cartridge. Wells # 3–7 contained wash buffer # 1 (600 μL and 250 μL), wash buffer # 2 (600 μL and 250 μL), and wash buffer # 3 (250 μL). Well # 8 contained an elution buffer (60 μL). Each pre-processed sample was run in triplicate. To ensure the quality of the work and to check for contamination, molecular-grade water was used for extraction during each run of the machine, instead of sample, and is considered the machine control (“Tx_μT_CTL” or “YNP_μT_CTL”).

The μTitan system was operated using a laptop computer (connected with a USB cable), loaded with an open-source software called Repetier-Host (Hot-World GmbH & Co., Willich, Germany) that uses G-code based programming commonly used for 3D printing and CNC milling. The G-code written by AI Biosciences for μTitan, was based on that written for a standard 3D printer, which provides instructions on the movement, temperature, and amount of material to be deposited. Since this is pre-programmed, a user just needs to click on the “print” button icon to start the automated extraction process.

Once the program started, the extraction tip comb (driver) magnet picked up the extraction tip magnet inside the cartridge box using a magnetic coupling mechanism and moved it to the subsequent wells of the cartridge. The NA bound magnetic particles attached to the base of the extraction tip were then washed sequentially in wash buffer # 1 and # 2, twice for 2 min each, and wash buffer # 3 for 30 s. Next, the magnetic particles were air-dried for 4 min. When the air-drying process began, the heater strip at the bottom of the elution well of the cartridge was heated to bring the elution buffer temperature to ∼70°C by a cartridge heater commonly used as the extruder of a 3D printer. This extruder heater’s function was also controlled by the program, and the temperature was precisely monitored with a thermistor. The NAs captured on the magnetic particles were eluted for 5 min in 60 μL of elution buffer. After elution, the magnetic particles were captured again by the extraction tip and moved back to well # 1, leaving only the eluate in the elution well (well # 8). The eluate was collected and stored in a 500 μL tube for future analysis. The μTitan system has the capability of processing 6 samples in parallel.

### Maxwell^TM^ Processing

Parallel extractions of the pre-processed samples were carried out on the Maxwell^TM^ automated extraction system (Promega Corporation, Madison, WI, United States) using a DNA kit (AS1520) and the “Maxwell^TM^ RSC blood DNA” run program. Briefly, 100 μL of the preprocessed samples were added to the cartridge and placed on the cartridge holder tray, and the DNA was eluted in 60 μL of elution buffer. Each pre-processed sample was run in triplicate. To ensure the quality of the work and to check for contamination, molecular-grade water was used for extraction during each run of the machine, instead of sample, and is considered the machine control (“Tx_Max_CTL”). The purified DNA from both machines was stored at −20°C until further analysis.

### Qubit Quantification and Quantitative PCR (qPCR)

Samples were quantified using two methods: the Q32854 Qubit^TM^ dsDNA HS Assay (ThermoFisher, Waltham, MA, United States) and qPCR using a standard curve SYBR green method using two control DNA standards. Qubit assay was performed using a 2 μl aliquot of DNA mixed with 198 μl of the dsDNA HS reagent and read on a Qubit 4 spectrofluorometer.

qPCR was performed on replicate 1 μl samples of 1:10 dilution of test DNA using the PerfeCTa SYBR Green SuperMix (95054 Quanta Bio, Beverly, MA, United States) using primers recommended by the Earth Microbiome Project -515 Forward GTGYCAGCMGCCGCGGTAA (Parada et al., 2016) and 806 Reverse GGACTACNVGGGTWTCTAAT (Walters et al., 2016). Standard curves were generated from two separate microbial reference control DNA, including (1) purified genomic DNA (gDNA) from the WCMR that had been extracted with Qiagen and (2) The ABRF/ATCC^®^ MSA-3001 gDNA (ATCC, Manassas, VA). Both standard curves were generated from 2, 0.2, 0.02, 0.002, and 0.0002 ng/μl of DNA. qPCR thermocycling was performed in 25 μl reactions on an ABI 7900HT (Applied Biosystems, Foster City, CA, United States) using the following three-step program: Denatured at 95°C -3 min, 40 cycles of PCR (95°C -15 s| 54°C-60 s| 72°C -60 s), followed by a standard dissociation curve (95°C-15s| 60°C-15s| 95°C-15s). Primers were included at a final concentration of 0.1 μM and low ROX as an internal reference.

### DNA Molecular Weight Analysis (Fragment Length) Using Bioanalyzer

DNA fragment size analysis (molecular weight) of the extracted DNA was evaluated using the Agilent Bioanalyzer 2100 with the High Sensitivity DNA chip (5067-4627) and the Advanced Analytical Technologies Fragment Analyzer 5200 with the HS Large Fragment 50kb Kit (DNF-493-0500) according to the manufacturer’s instructions.

### Illumina Sequencing

#### Library Preparation

Whole genome shotgun sequencing libraries were synthesized using Nextera XT reagents (Illumina Corp, San Diego, CA, United States) from either 3 ng of total DNA for samples > 0.5 ng/μl or 6 μl input for samples below 0.4 ng/μl. Final libraries were assessed for quality using the Qubit^TM^ dsDNA HS Assay and Agilent Bioanalyzer 2100 assessment using the DNA high sensitivity chip (5067-4626 Agilent Corp., Santa Clara, CA, United States). All samples, including positive and negative controls, were pooled by combining the high input (>0.5 ng/μl), using 2.5 ng of library, with the low input, using 0.25 μl, to prevent over representation of low input samples. Pooled hybridization cocktails were clustered and sequenced using a rapid run SR flow cell (GD-402-4002) for 150 bases on the Illumina HiSeq 1500.

#### Metagenome Sequence Data Processing

Adapter sequences and low-quality reads (Phred score < 20 across the entire length of the read) were removed with Trimmomatic and reads that were shorter than 80 bp after quality control trimming were discarded. Post-processing resulted in 53,549,976 high-quality reads. DIAMOND (Buchfink et al., 2015) and the weighted lowest common ancestor (LCA) algorithm of MEGAN6 (Huson et al., 2007) (Settings of minScore = 50, maxExpected = 0.01, topPercent = 10, and minSupportPercent = 0.01) was used to cluster high-quality filtered reads to taxonomic and functional levels. BLAST hits of ≥ 20 amino acids and ≥ 90% similarity were collected and used for taxonomic and functional assignment using the NCBI taxonomy database which contains over 6.6 × 10^5^ reference sequences (Sayers et al., 2009), and NCBI-NR protein sequence database which consists of entries from GenPept, SwissProt, PIR, PDB, and RefSeq, were used with MEGAN6 (Huson et al., 2007) to perform the taxonomic and functional binning of the metagenomic reads.

### Oxford Nanopore Sequencing

Oxford Nanopore libraries were synthesized for all samples from input ranges of BDL (below detection limit of the Qubit) up to 5 ng for higher input samples using the Rapid PCR barcoding kit (RPB004 Oxford Nanopore Technologies, Oxford, United Kingdom) with the following modifications: high input samples were amplified using the standard method of 14 PCR cycles, while samples that were below the detection limit of Qubit were amplified using 22 PCR cycles. Barcoded libraries were pooled equally at 1 fmol each for the high samples, while the BDL samples were pooled at the maximum volume allowable for library input. Sequencing was performed using the MK1B MinIon with 9.4 flow cell yielding > 1 million reads of 2 Gbases with average read lengths of 2–8 kb. All quality control statistics were within specification as outlined by Oxford Nanopore.

Microbial reference controls were also sequenced on the Oxford Nanopore sequencer, including the purified gDNA from the Qiagen Benchmark sample. Rapid PCR barcoded libraries were synthesized from input concentration identical to the qPCR standard curve at 6, 0.6, 0.06, 0.006 ng. This titration was performed to determine the detection limits, linearity, and efficiency for ultra-low input samples.

Oxford Nanopore data was analyzed using the WIMP (What’s In My Pot) module of the EPI2ME software (Oxford Nanopore Technologies, Oxford, United Kingdom), and raw read count data was exported as CSV data for manual parsing and enumeration using Microsoft Excel.

Nanopore detection and sequencing of the low biomass samples revealed negligible concentrations above background. This is not surprising since the input concentrations were below the recommended input for the Rapid PCR barcoding kit. For these reasons, Nanopore data generated from low biomass samples were not included.

### Data Analysis

The Illumina sequencing data was analyzed in R version 3.3.1 using the Hmisc, ggplot, compositions and cluster packages. For the PCA plot, the data was first center log ratio (clr) transformed and the Euclidian distances plotted. K-means clustering was used to determine the number of distinct groups and the samples that belonged to these groups.

DNA yield and qPCR graphs were generated in Prism version 7.0. Statistical analyses were executed using one-way analysis of variance (ANOVA) and then using the Benjamini- Hochberg false discovery rate (FDR) multiple test correction. Statistical significance was set at *P* < 0.05.

## Results

### Testing and Validation of the μTitan System

**FIGURE 4:**
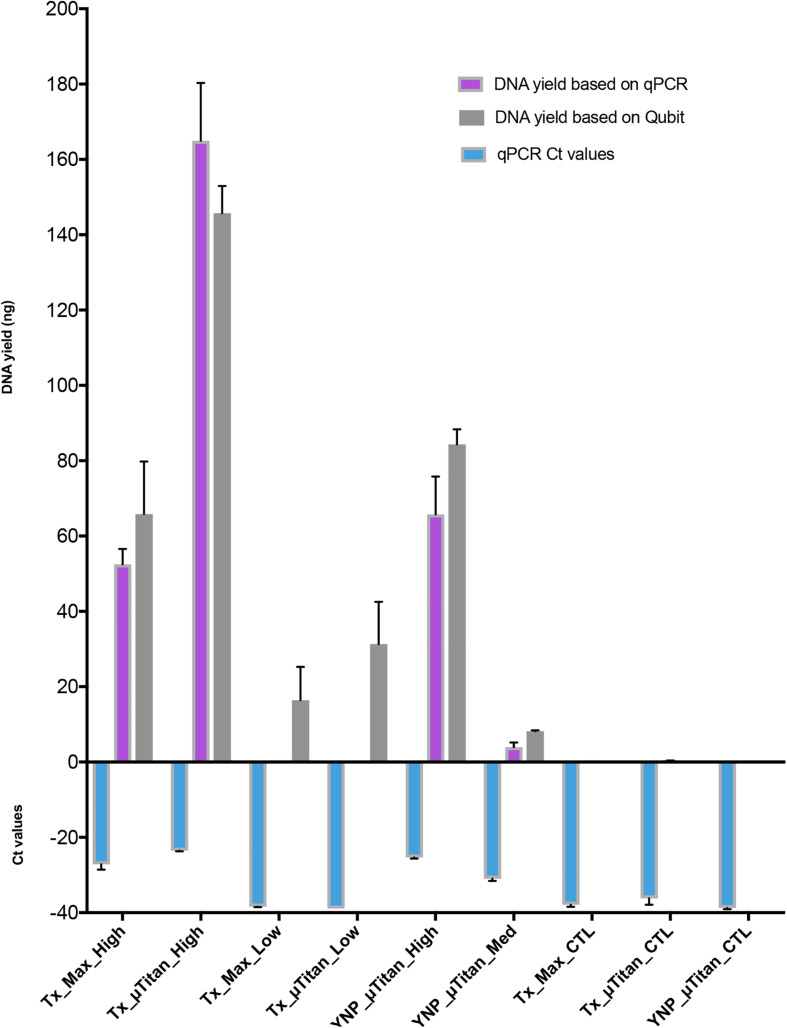
DNA quantification of the extracted WCMR. DNA extraction of the WCMR was performed by both Maxwell^TM^ and μTitan systems in the lab of AI Biosciences (Texas; “Tx”) or in the field by μTitan (Yellowstone National Park; “YNP”). The qPCR Ct values are shown by the blue bars. Known amounts of WCMR DNA, extracted by Qiagen, was used to generate a standard curve during qPCR, by which DNA yield in ng could be calculated (purple bars). NB: The lower the Ct value, the higher the amount of DNA in that sample. The DNA yield was also measured by Qubit (gray bars). Different amounts of biomass; high, medium, or low were extracted with both Maxwell^TM^ and μTitan systems. High biomass samples represent inputs of approximately 10^7^ cells, low approximately 10^4^, and medium approximately 10^6^. As can be observed, the μTitan system generated higher DNA yield than the Maxwell^TM^ system for both high and low biomass samples. Also, the μTitan system in the field produced comparable results to that obtained in the lab (YNP_μT_High vs Tx_μT_High).

A whole cell microbial reference standard, that contained intact microbial cells (Table 1) was extracted using both μTitan and Maxwell^TM^ instruments in a laboratory setting. High biomass (10^7^ cells) and low biomass (10^4^) cellular concentrations were used in the validation tests. WCMR standard was extracted using both the μTitan and Maxwell^TM^ instruments in a laboratory setting. The composition of the WCMR (Table 1) contains fully intact microbial cells of Gram +/−, high and low GC, and a range of morphologies. Two cellular concentrations were used in the extraction tests of the two instruments representing high biomass (10^7^ cells) and low biomass (10^4^ cells) inputs and subject to pretreatment with a cell wall digesting enzyme mix (Metapolyenzyme) and mechanical bead beating. After pretreatment, replicate 100 μl aliquots were used as inputs to both instruments. Following extraction, the yield of recovered DNA was determined using a Qubit spectrofluorometer and qPCR (Figure 4). These results indicate that the μTitan system produced higher DNA yields, with both the high biomass (“Tx_x_High”) and low biomass (“Tx_x_Low”) samples; however, only the high cellular concentrations were statistically different.

**FIGURE 5:**
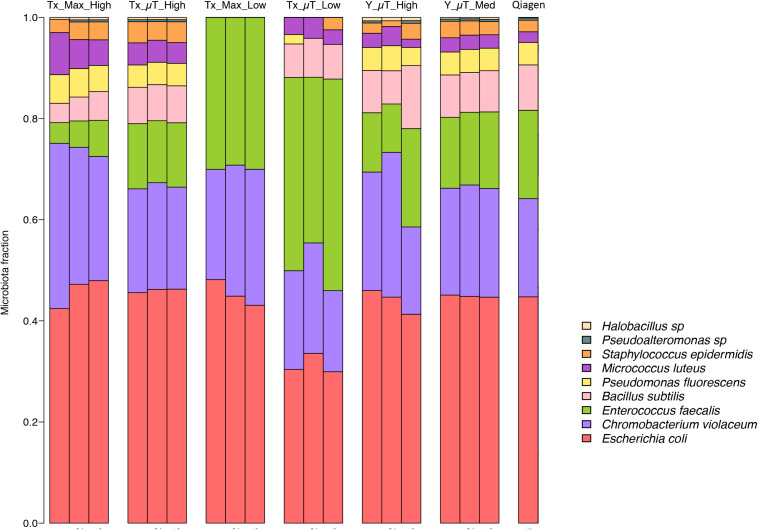
Relative abundances of the nine bacteria present in the WCMR. The WCMR DNA that was extracted by the Maxwell^TM^ and μTitan systems was sequenced on the Illumina HiSeq platform, and the relative abundances of the nine bacteria present in the WCMR shown in this bar graph. Each bar represents a sample, and each colored box represents the proportion of a particular organism in that sample. For comparison, the WCMR was also manually extracted with Qiagen, sequenced, and plotted. The high and medium biomass samples were able to detect all nine organisms, in the same relative abundances, which was also comparable to that observed with Qiagen extraction. The low biomass samples did not detect all bacteria in the WCMR standard, but the μTitan system detected more than the Maxwell^TM^. A complete list of the bacteria detected and their counts are summarized in Table 3.

Shotgun metagenomic sequencing was performed on all samples using the Illumina HiSeq 2500 platform to determine whether the isolated DNA could be used to prepare successful sequencing libraries and obtain high-quality sequencing results. Table 3 shows the WCMR members detected in each sample from both extraction instruments and their respective counts. The bar graph in Figure 5 shows the proportion of these nine bacteria in each sample and also includes the proportion of bacteria detected when a manual Qiagen QIAquick extraction (Qiagen Benchmarking Control) was performed in the lab with high efficiency pre-processing steps added. Both Table 3 and Figure 5 show that DNA from all nine bacteria could be isolated from the high biomass samples extracted with either the μTitan (“Tx_μT_High”) or the Maxwell^TM^ instrument (“Tx_Max_High”). For the low biomass samples, the μTitan system had increased performance over the Maxwell^TM^ and from the DNA extracted, six out of nine bacteria were identified (“Tx_μT_Low”), while from the Maxwell^TM^ extracted DNA, only three out of nine bacteria were identified (“Tx_Max_Low”). The proportions of identified bacteria in the high biomass samples were similar between the two instruments as well as for the Qiagen Benchmarking Control (Figure 5).

**TABLE 3:** Illumina sequencing read counts of the bacterial species present in the whole cell microbial reference standard detected from the μTitan and Maxwell^TM^ systems in both a laboratory and field setting.

Microbial taxa	# sequences retrieved with high biomass samples in a lab (Texas)						# sequences retrieved with low biomass samples in a lab (Texas)						# sequences retrieved with high and medium biomass samples processed with μTitan in the field (YNP)					
	
	Maxwell^TM^ (Tx_Max_High)			μTitan (Tx_μT_High)			Maxwell^TM^ (Tx_Max_Low)			μTitan (Tx_μT_low)			YNP_μT_High			YNP_μT_Medium		
						
	rep 1	rep2	rep3	rep1	rep2	rep3	rep1	rep2	rep3	rep1	rep2	rep3	rep1	rep2	rep3	rep1	rep2	rep3
*Escherichia coli*	2,88,592	3,17,753	3,26,481	2,81,112	2,79,525	2,73,380	1,027	421	561	1,955	1,310	2,171	3,18,603	3,34,962	3,61,581	2,42,269	2,50,667	2,70,935
*Chromobacterium violaceum*	2,22,242	1,82,297	1,67,347	1,26,575	1,27,509	1,19,165	465	243	350	1,255	852	1,162	1,49,285	1,64,426	1,73,854	1,23,261	1,60,574	1,13,173
*Enterococcus faecalis*	27,762	34,981	48,527	79,526	74,161	75,180	641	274	391	2,458	1,278	3,034	99,057	1,07,369	1,22,557	61,701	53,609	1,27,570
*Bacillus subtilis*	26,060	31,835	38,835	44,318	43,126	43,181	–	–	–	426	301	496	59,178	58,916	66,009	44,056	36,867	81,776
*Pseudomonas fluorescens*	38,503	37,911	35,184	27,282	26,703	26,217	–	–	–	120	–	–	32,041	33,889	35,996	23,954	27,818	23,405
*Micrococcus luteus*	56,593	38,511	34,463	26,957	26,382	24,319	–	–	–	218	161	210	19,898	21,191	21,476	14,784	21,192	10,843
*Staphylococcus epidermidis*	17,675	23,449	24,162	25,813	22,314	24,302	–	–	–	–	–	178	22,921	20,992	21,141	10,689	6,422	20,576
*Pseudoalteromonas* sp.	–	2,793	2,873	2,474	2,399	2,500	–	–	–	–	–	–	3,227	3,213	3,503	2,234	–	3,492
*Halobacillus* sp.	2706	3298	3182	2775	2621	2618	–	–	–	–	–	–	3590	3754	4177	2341	1966	3132

The μTitan system was further evaluated in a Phase II study at Yellowstone National Park (YNP) to test its performance in a remote setting with limited resources. The Maxwell^TM^ system was not included in this field study due to its size, weight, power, and calibration requirements. In this phase of testing, two concentrations of the WCMR were used; a high biomass sample (“YNP_μT_High”) similar to that used in the laboratory test (“Tx_μT_High”) and a medium biomass sample composed of a 10-fold dilution of the high biomass sample (“YNP_μT_Med”, 10^6^ cells).

The resulting DNA yield for the high biomass sample, as measured by qPCR was comparable to the yield obtained in the laboratory tests (“Tx”), indicating the μTitan system is able to produce consistent results even when used in a remote setting (Figure 4). Results of the Illumina-based shotgun metagenomic sequencing were also consistent with the laboratory tests in that all nine bacteria in the WCMR were detected in both the high and medium biomass samples (Table 3), in the same proportions as that observed in the “Tx_μT_High” and Qiagen benchmark sample (Figure 5).

As is expected from any shotgun metagenomics sequencing run, there will be some measure of contaminant reads that come from extraction and sequencing processes (the “kitome”), and this dataset was no exception. The heatmap in Supplementary Figure S1 shows the bacterial contaminants (i.e., any species that did not belong to one of the nine bacteria in the WCMR along with their counts) in all samples.

**FIGURE 6:**
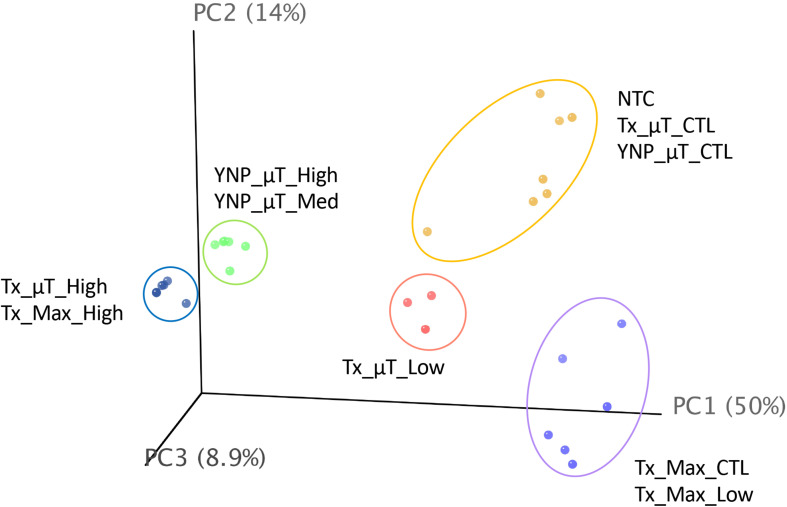
Principal coordinate analysis of shotgun metagenomic sequencing data generated from the Illumina platform. Results for each sample were plotted on a 3-D, 3-axis plane, representing 73% of the variation observed amongst all samples. Each point represents a sample, and the closer a sample is to another, the more similar their microbiome composition (diversity and abundance). There were 5 distinct groups in this dataset, represented by the colored ellipses, which were determined by unsupervised k-means clustering.

A PCA plot (Figure 6) comparing the diversity and relative abundances of the species present in the samples and controls (both WCMR bacteria and contaminants) showed that the high and medium biomass samples had similar microbiomes, regardless of whether they were extracted with the μTitan system (green points) or with the Maxwell^TM^ instrument (blue points), and were distinct from the kitomes and machine controls. With the low biomass samples, the μTitan samples (Tx_μT_Low, pink points) were distinct from their respective controls, but this was not the case with the Maxwell^TM^ samples (Tx_Max_Low, purple points), which were indistinguishable from their respective controls. Additionally, the PCA plot indicates that replicate extractions with the μTitan system were consistent and produced comparable results (replicates group together on the plot). More importantly, the plot shows that the field-operated μTitan system (YNP_μT_High) and lab-operated system (Tx_μT_High) produced similar results.

In addition to the Illumina HiSeq data, shotgun sequencing was also performed on the high and medium biomass samples using Oxford Nanopore Technologies’ MinIon MK1B. The importance of including Nanopore data in this study was to provide an alternative sequencing technique that would avoid any Illumina Nextera short-read sequencing biases due to GC content, to enable detection using long reads with an alternative library synthesis that may influence taxonomic classification due to genome complexity and long genomic repeats (e.g., *Pseudoaltermonas*), and to determine how DNA extracted from μTitan would sequence on the MinION MK1B as this is the sequencing platform that is currently being used on the ISS and in the field. Although read depth, read length, and Q-Score are different between Nanopore and Illumina sequencing technologies, the organisms belonging to the WCMR were just as easily detected and classified with the MinION using DNA isolated from both the Maxwell^TM^ and μTitan systems, in both the laboratory and field settings. Tabulation of organisms in each sample belonging to the WCMR was represented at the Genus level on raw data normalized to 50,000 reads to allow for direct comparison between samples (Table 4). These results indicate that, when compared to the Maxwell^TM^ instrument, the μTitan system generated a marginally greater number of reads of *Staphylococcus* and *Bacillus*, and an even greater number of *Enterococcus*, all of which are Gram-positive bacteria. On the other hand, more reads were observed by the Maxwell^TM^ instrument for the Gram-negative bacteria, *Escherichia* and *Pseudomonas*. These trends observed with the Nanopore data are consistent with that observed with the Illumina data.

**TABLE 4:** Enumeration and detection of genera belonging to the whole cell microbial reference control using Oxford Nanopore Sequencing.

Microbial taxa	Number of reads for high biomass						Number of reads for samples						Benchmark control: DNA			
	samples (Texas lab trials)						extracted using the μTitan in the						extracted with Qiagen			
							remote field location (YNP)									
			
	Maxwell^TM^			μTitan			YNP_μ			YNP_μ			Benchmark DNA Control			
	(Tx_Max_High)			(Tx_μ T_High)			T_High			T_Medium			DNA (ng Input)			
					
	rep 1	rep2	rep3	rep1	rep2	rep3	rep1	rep2	rep3	rep1	rep2	rep3	6.0	0.6	0.06	0.006
*Escherichia*	17454	16576	17,445	12,727	13,265	13,598	15442	14494	15710	18035	10155	18087	17420	10799	658	20
*Chromobacterium*	1998	1280	1,651	941	1510	1051	1085	1090	1447	1223	2501	1216	1618	1810	121	0
*Enterococcus*	918	1015	1,002	2766	3024	3063	2726	3339	2762	1370	2808	1378	2132	2991	142	11
*Bacillus*	4659	4883	5,148	5715	6989	5757	8440	8465	8138	8828	8942	8201	8823	8841	434	21
*Pseudomonas*	7428	4730	6,051	3068	4336	3470	3728	3545	4818	6861	6829	7543	7259	6770	585	35
*Micrococcus*	84	73	96	28	62	28	17	18	29	18	123	26	46	50	1	0
*Staphylococcus*	6555	10459	8,060	9891	9280	13032	5114	6098	5050	3289	5344	3727	2553	2843	173	11
*Pseudoalteromonas*	5850	6471	6,006	6401	6918	6694	9501	9123	8029	5880	7770	5304	6479	7745	350	19
*Halobacillus*	105	116	113	125	104	111	125	101	89	99	68	109	78	119	2	0

*Data normalized to 50,000 reads for sample.*

**FIGURE 7:**
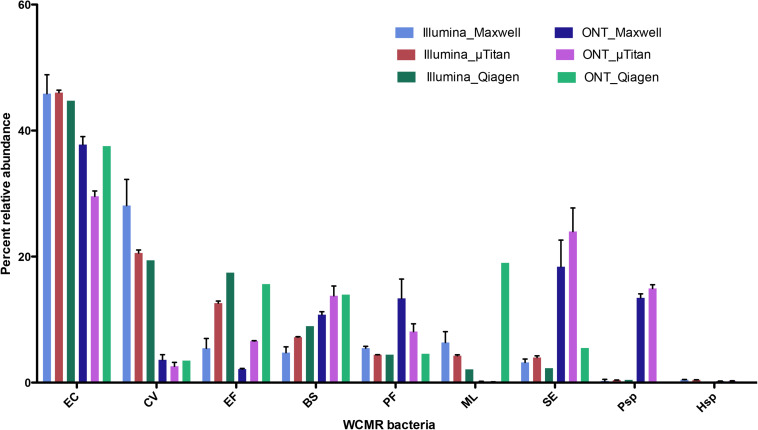
Comparison of Illumina Nextera XT data to Oxford Nanopore Rapid PCR barcoding data. Percent abundance of the WCMR bacteria detected by either Illumina short read versus Oxford Nanopore long reads, from DNA isolated from either the Maxwell^TM^ or μTitan system were compared with boxplots. The height of the box represents the average of triplicate DNA extractions from just the high biomass samples. The error bars represent the standard deviation. Generally, for high biomass samples, the μTitan system performs as well as the Maxwell^TM^, and in this case, Illumina short reads worked better for certain genera (*Chromobacterium*, *Enterococcus*, and *Micrococcus*), while the Nanopore long reads worked better for others (*Pseudomonas*, *Pseudoaltomonas*, *Bacillus*, and *Staphylococcus*).

The proportions of the WCMR were compared between Illumina and Nanopore data for the purpose of detecting any bias inherent to the sequencing method (Figure 7). These data indicates that Illumina short-read sequencing using Nextera XT libraries has increased ratios of detection for *Chromobacterium*, *Enterococcus*, and *Micrococcus*, while the Oxford Nanopore rapid PCR data has an increase detection ratio for *Pseudomonas*, *Pseudoalteromonas*, *Bacillus*, and *Staphylococcus*.

The extracted DNA from the high and medium biomass samples were also analyzed using an Agilent Bioanalyzer 2100 to determine the DNA fragment length. For both the Maxwell^TM^ and μTitan instruments, the fragment length amongst the replicates were very consistent, with the μTitan system producing slightly larger DNA fragments (average length: 6,625 bp) compared to the Maxwell^TM^ system (average length: 5,604 bp). These results were confirmed with a subsequent analysis using a high-sensitivity assay with the Advanced Analytical Fragment Analyzer 5200 (data not shown), which was closer to the fragment length obtained by using a Qiagen Benchmark control manual extraction (average length: 7,912 bp) (Table 5; Supplementary Figure S2).

**TABLE 5:** Fragment length and molecular weight results for instrument extracted samples and reference DNAs using the Agilent Bioanalyzer 2100.

DNA Orgin	Replicate	Range (MW)		Mean	Average		
				
		Low	High		Low	High	Mean
Tx_Max_H	1	527	19642	5484			
	2	580	15785	5817	581	17324	5604
	3	636	16544	5511			
Tx_μT_H	1	510	20213	6337			
	2	543	27570	6463	534	24126	6405
	3	550	24595	6415			
YNP_μT_H	1	200	23683	6683			
	2	527	29271	6977	498	25187	6845
	3	766	22608	6876			
YNP_μT_Medium	1	1035	22040	9412			
	2	1142	19653	9058	1060	23274	9341
	3	1003	28131	9554			
Qiagen extracted WCMR	1	191	41703	8025			
	2	312	34104	7924	234	34924	7912
	3	200	28965	7788			

### Microgravity Compatibility Testing of the μTitan System

While the μTitan cartridges used in these experiments were not designed for microgravity compatibility, the newest set of cartridges have been designed to exploit surface tension properties to prevent the unwanted release of fluids (Weislogel et al., 2009, 2011). These cartridges accommodate fluids and a magnetic probe and allow the fluids to preferentially remain at the base of the cartridge well, discouraging wetting of the upper portion of the well, thus preventing liquids from floating free when used in microgravity (Weislogel et al., 2009, 2011). The microgravity fluid physics behind this design comes from the same team who engineered the “space cup” in 2015, which allowed astronauts on the ISS to drink hot espresso from an open coffee cup for the first time (NASA, 2015). Several 2.1s drop tower tests have been performed on the μTitan system (used in the current study) and the newly designed cartridges, and during these low-gravity tests, the fluids remained at the bottom of the cartridge wells, as designed (Supplementary Figure S3), indicating its compatibility with use on the ISS.

## Discussion

This manuscript describes the development and validation of the μTitan system, a high-performance, automated, and programmable nucleic acid extraction platform designed for use in both microgravity and Earth-based assays. The system employs the use of magnetic beads, a probe, and specialized chemistry for the isolation of high-quality nucleic acids for downstream analyses such as qPCR and NGS. This has all been made possible by the low cost and high precision of consumer-level 3D printers (Coakley and Hurt, 2016).

The results of the present study have shown that the μTitan system can isolate high molecular weight DNA that can be used for qPCR and high-quality NGS libraries for both Illumina and Oxford Nanopore platforms regardless of the assay. The instrument run time could extract high-quality DNA from the WCMR in 20 min (μTitan) compared to 40 min (Maxwell^TM^) with the comparative method used in this study. Additionally, the system produced higher DNA yields (Qubit and qPCR) and slightly higher molecule weight DNA fragments (Agilent Bioanalyzer) than the Maxwell^TM^ system. Both the Illumina and Nanopore sequencing platforms showed that the μTitan system is able to isolate DNA from Gram-positive organisms better than the Maxwell^TM^ system. The μTitan system also detected more WCMR bacterial species in the low biomass input and was also less influenced by processing and sequencing contaminants than the Maxwell^TM^ system. The potential for cross contamination when multiple samples are run simultaneously was also examined. Using both high biomass and low biomass samples as input and running them alongside negative control samples (i.e., water as the input), no evidence of carryover from sample to negative control was observed when tested with Qubit and qPCR (data not shown).

While great strides have been made by NASA in the past five years to allow for molecular biology to be performed on the ISS, with WetLab-2 (Parra et al., 2017), miniPCR^TM^ (Boguraev et al., 2017), Razor-EX (Cherie et al., 2013), and MinION (Castro-Wallace et al., 2017), there exists a need for an automated, multiple sample processing system for the ISS for downstream omics analyses. The development of the μTitan system, which can extract nucleic acids (and in the future, protein) from a variety of environmental (manuscript under preparation) and human (Chan et al., 2016a, c) samples now allows for the ability to provide end-to-end processing of biological samples from raw material to purified nucleic acids, all while in orbit. The advantage of the μTitan system over previous ISS studies that have used mechanical bead beating or thermal lysing to extract nucleic acids is that the μTitan system works for various sample types (i.e., environmental or human) of complex microbial communities, for both high biomass and low biomass input, and not only for high concentration of pure bacterial cultures, which the previous methods were tested on. The μTitan system is also automated and requires less crew time than previous protocols. The dedicated clean-up and washing steps allow for cell debris to be removed, leading to higher purity nucleic acids, allowing for better sensitivity for low biomass samples and overall better detection of the diversity within a sample. Most current automated systems on the market, as well a manual extraction kits suggest the inclusion of an optional bead beating step if the user so desires. In addition to bead beating our team also included the use of metapolyenzyme (MPZ), as it has been shown for even greater lysis efficiency than just bead beating alone. The reagents within the μTitan system would be able to extract DNA without bead beating and MPZ treatment but for low biomass samples, when one needs to extract as much DNA as possible, these additional lysis steps are important. An additional pre-processing module is being developed where enzymatic digestion and bead-beating will be performed which will be compatible for the μTitan system and will be in an enclosed system to prevent aerosols on the ISS. The μTitan system is useful for most the microorganisms but for hardy microorganisms such as spore-formers and actinobacteria, a pre-processing step is needed and thus has been included in the design, if needed.

NASA performs routine microbial monitoring on the ISS, but the culture-based methods that are currently used do not provide a comprehensive assessment of what microbes are actually present. For this reason, NASA is actively working toward increasing their monitoring capabilities by testing different *in situ* protocols for DNA extraction that can be used for downstream qPCR on the ISS (Cherie et al., 2013). The advantage of the μTitan system over the other methods currently being tested is that, unlike a minimum of 10^5^ cells per μL that is needed to positively detect microorganisms, a μTitan output concentration of 200 cells/μl is able to be successfully analyzed by NGS and qPCR because of the chemical processes involved in the extraction workflow that increase DNA yield and purity. Furthermore, high-quality DNA from six samples can be obtained in 45 min, with only 15 min of crew time, allowing for high-throughput sample processing aboard the ISS as opposed to manual, laborious, and time-consuming pre-existing methods.

The ability to perform high-throughput sample processing onboard the ISS followed by NGS makes it possible for astronauts to know what microbes and their properties are in the environment, at any given time. This is significant when you consider that omics analyses on ISS samples (performed on Earth) have detected novel organisms and/or those with unique properties (Checinska Sielaff et al., 2017; Romsdahl et al., 2018, 2019; Singh et al., 2019; Urbaniak et al., 2019). Furthermore, many terrestrial species that have been sent from Earth to grow on the ISS have become more virulent and antibiotic resistant, and have formed more biofilms (Yamaguchi et al., 2014), all of which are properties that can affect the health of astronauts and the stability of the spacecraft. The μTitan system will also be instrumental for crew monitoring. Having the μTitan system on the ISS to process samples and generate data provides the capability to immediately detect and measure several biomolecules related to the physiological and immunological effects of living in space. For example, viral presence during spaceflight is a useful *in vivo* biomarker of immunodeficiency in astronauts (Mehta et al., 2004; Pierson et al., 2005) and was recently demonstrated to positively correlate with immune alterations in astronauts after spaceflight (Mehta et al., 2013). The ability to quantify viral load in astronauts in space, in real time, will provide an efficient means to monitor immune function and to allow for proper countermeasures to be implemented, such as vaccination strategies, prophylactic antibiotics, or stress reduction therapies (Kiecolt-Glaser et al., 2010; Kiecolt-Glaser et al., 2014).

## Conclusion

The μTitan instrument is a compact, portable, robust, energy-efficient device that allows for streamlined and consistent nucleic acid extractions that requires minimal human labor. It provides the ability to perform complex sample processing on the ISS to gain real-time information from environmental and human samples. While this study has validated the instrument for DNA isolation, it has been validated previously for successful extraction of RNA (Chan et al., 2016b) and thus can be used to process samples for microbiome, metagenome, transcriptome, and virome analyses on the ISS and possibility on other deep space missions. The characteristics that make it advantageous for space travel also make it suitable for remote settings here on Earth. Whether the μTitan system is used on the ISS or on Earth, it provides high-quality nucleic acid material for functional genomics, microbial monitoring, and detection of biological signatures related to human health and engineering systems.

## Data Availability Statement

The datasets presented in this study can be found in online repositories. The names of the repository/repositories and accession number(s) can be found in the article/Supplementary Material.

## Author Contributions

KV designed the concept and objectives of the study with all authors of the manuscript. CU wrote the manuscript, helped design the study, critically analyzed the data, generated all the figures and performed statistical analyses, wrote the R scripts to analyze the NGS data, and processed the WCMR at YNP. SW invented μTitan and performed all initial studies prior to this validation study, and processed the WCMR in Texas and in YNP. ST helped develop the WCMR; performed quality control on the WCMR before using it for validation studies; and performed NGS, qPCR, Qubit, and bioanalyzer analyses. AA performed initial validation of μTitan and processed the WCMR in Texas and YNP. BL, CP, and JW participated and performed experiments in Texas and YNP. JW and NS processed the raw Nanopore sequencing data and raw Illumina data and helped design the study. JW, BP, and DS obtained safety protocol for using scientific expeditions at YNP with appropriate science permit provided by the YNP and cleared by JPL, Permit #YELL-2019-SCI-5480. BP and DS participated in sample collections and processing in the YNP and also processing in the MSU lab. RJ carried out the microgravity compatibility study, and FK carried out crew procedure development of the μTitan system. All authors critically reviewed the manuscript.

## Disclaimer

Reference herein to any specific commercial product, process, or service by trade name, trademark, manufacturer, or otherwise does not constitute or imply its endorsement by the U.S. Government or the Jet Propulsion Laboratory, California Institute of Technology © 2020 California Institute of Technology. Government sponsorship acknowledged.

## Conflict of Interest

AA and BL were employed by AI Biosciences, Inc. SW is the co-founder of AI Biosciences, Inc. RJ was employed by IRPI LLC. The remaining authors declare that the research was conducted in the absence of any commercial or financial relationships that could be construed as a potential conflict of interest.

## Supplementary Material

FIGURE S1 Summary of sequences detected by shotgun metagenomic sequencing not represented in the WCMR standard. Heatmap of counts of non-WCMR-represented species in the 18 samples and 10 controls. These non-represented species indicate contaminants from sample processing, including extraction reagents, library synthesis, or data analysis artifacts. Each sample represents the average of sample extraction triplicates.

FIGURE S2 Fragment size and molecular weight determination of extracted DNA. Agilent Bioanalyzer 2100 data for samples analyzed with the DNA HS chips for Maxwell^TM^ system extracted samples (laboratory setting in Texas) and μTitan system samples (laboratory setting in Texas and field setting in YNP).

FIGURE S3 Drop tower test. A 2.1 s drop tower test was performed on the μTitan system and shows the fluid reorientation following a step transition from 1-g to low-g. The case on the left shows a top-down view of fluid reorientation after entering low-g. The case on the right shows a well with a probe partially submerged. A minor change in fluid orientation occurs after entering low-g, but the fluid stays in the preferred position, as desired. Note: Arrows indicate the direction of g prior to entering low-g.

TABLE S1 Table of read counts generated from Illumina HiSeq sequencing. The first nine organisms are the ones present in the WCMR.
