# Supplementary material for: Validating an Automated Nucleic Acid Extraction Device for Omics in Space Using Whole Cell Microbial Reference Standards
Source: Front Microbiol. 2020 Aug 21;11:1909. doi: 10.3389/fmicb.2020.01909 (PMC7472602; doi:10.3389/fmicb.2020.01909)

Figure S1

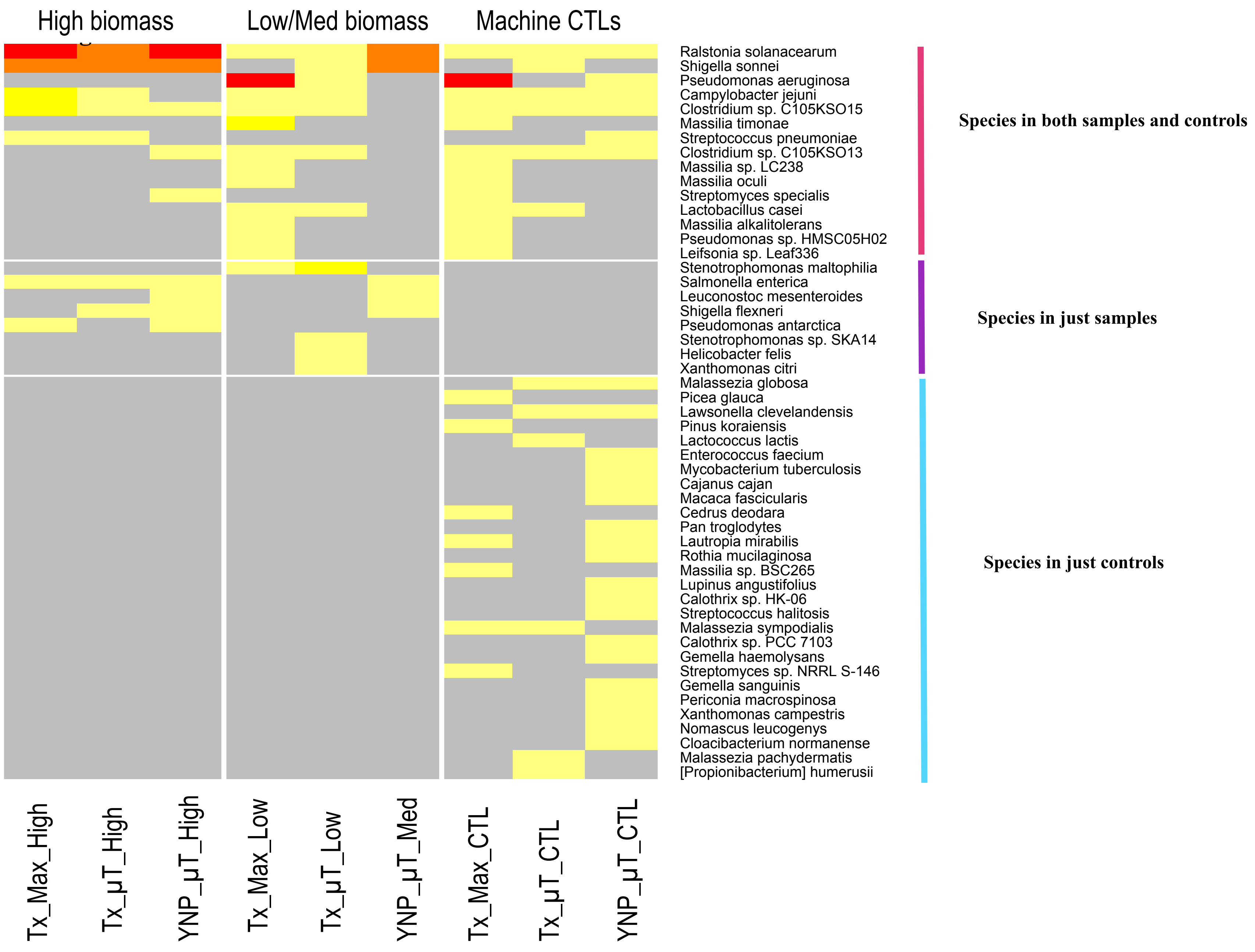
High biomass
Low/Med biomass
Machine CTLs
Ralstonia solanacearum
Shigella sonnei
Pseudomonas aeruginosa
Campylobacter jejuni
Clostridium sp. C105KSO15
Massilia timonae
Streptococcus pneumoniae
Clostridium sp. C105KSO13
Massilia sp. LC238
Massilia oculi
Streptomyces specialis
Lactobacillus casei
Massilia alkalitolerans
Pseudomonas sp. HMSC05H02
Leifsonia sp. Leaf336
Stenotrophomonas maltophilia
Salmonella enterica
Leuconostoc mesenteroides
Shigella flexneri
Pseudomonas antarctica
Stenotrophomonas sp. SKA14
Helicobacter felis
Xanthomonas citri
Malassezia globosa
Picea glauca
Lawsonella clevelandensis
Pinus koraiensis
Lactococcus lactis
Enterococcus faecium
Mycobacterium tuberculosis
Cajanus cajan
Macaca fascicularis
Cedrus deodara
Pan troglodytes
Lautropia mirabilis
Rothia mucilaginosa
Massilia sp. BSC265
Lupinus angustifolius
Calothrix sp. HK-06
Streptococcus halitosis
Malassezia sympodialis
Calothrix sp. PCC 7103
Gemella haemolysans
Streptomyces sp. NRRL S-146
Gemella sanguinis
Periconia macrospinosa
Xanthomonas campestris
Nomascus leucogenys
Cloacibacterium normanense
Malassezia pachydermatis
[Propionibacterium] humerusii
Species in both samples and controls
Species in just samples
Species in just controls
Tx_Max_High
Tx_µT_High
YNP_µT_High
Tx_Max_Low
Tx_µT_Low
YNP_µT_Med
Tx_Max_CTL
Tx_µT_CTL
YNP_µT_CTL

Tx_Max_H

Figure S2

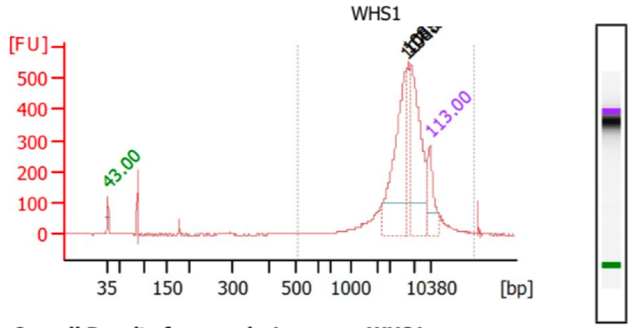

Peak table for sample 1 : WHS1

Peak		Size [bp]	Conc. [pg/µl]	Molarity [pmol/l]	Observations
1	◀	35	125.00	5,411.3	Lower Marker
2		5,587	281.12	76.2	
3		5,912	64.80	16.6	
4		6,237	278.89	67.8	
5	▶	10,380	75.00	10.9	Upper Marker

Overall Results for sample 1 : WHS1

Number of peaks found:	3
Noise:	0.6
Corr. Area 1:	3,767.8

Region table for sample 1 : WHS1

From [bp]	To [bp]	Corr. Area	% of Total	Average Size [bp]	Size distribution in CV [%]	Conc. [pg/µl]	Molarity [pmol/l]	Color
510	20,213	3,767.8	95	6,337	49.0	723.85	266.4	

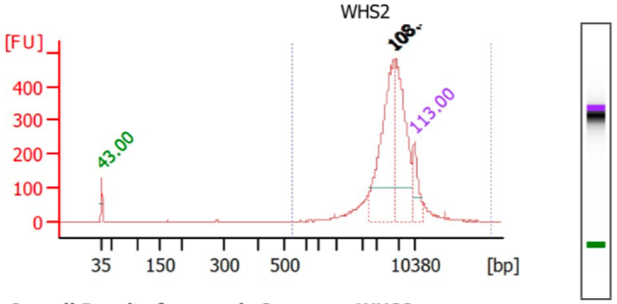

Peak table for sample 2 : WHS2

Peak		Size [bp]	Conc. [pg/µl]	Molarity [pmol/l]	Observations
1	◀	35	125.00	5,411.3	Lower Marker
2		5,875	375.19	96.8	
3		6,239	318.33	77.3	
4	▶	10,380	75.00	10.9	Upper Marker

Overall Results for sample 2 : WHS2

Number of peaks found:	2
Noise:	0.9
Corr. Area 1:	3,270.3

Region table for sample 2 : WHS2

From [bp]	To [bp]	Corr. Area	% of Total	Average Size [bp]	Size distribution in CV [%]	Conc. [pg/µl]	Molarity [pmol/l]	Color
543	27,570	3,270.3	99	6,463	52.3	801.18	287.8	

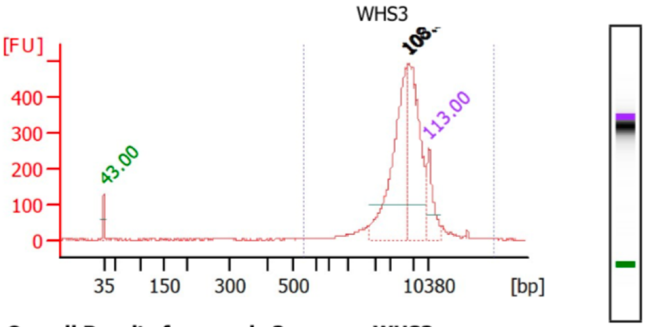

Peak table for sample 3 : WHS3

Peak		Size [bp]	Conc. [pg/µl]	Molarity [pmol/l]	Observations
1	◀	35	125.00	5,411.3	Lower Marker
2		5,903	359.39	92.2	
3		6,162	282.46	69.5	
4	▶	10,380	75.00	10.9	Upper Marker

Overall Results for sample 3 : WHS3

Number of peaks found:	2
Noise:	1.0
Corr. Area 1:	3,334.6

Region table for sample 3 : WHS3

From [bp]	To [bp]	Corr. Area	% of Total	Average Size [bp]	Size distribution in CV [%]	Conc. [pg/µl]	Molarity [pmol/l]	Color
550	24,595	3,334.6	99	6,415	50.7	700.16	252.9	

Tx_µT_H

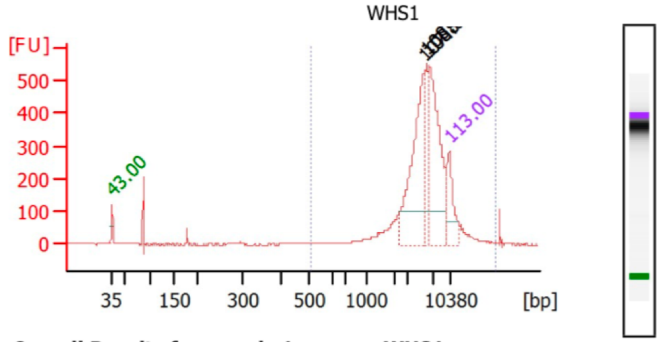

Overall Results for sample 1 : WHS1

Number of peaks found:	3
Noise:	0.6
Corr. Area 1:	3,767.8

Region table for sample 1 : WHS1

From [bp]	To [bp]	Corr. Area	% of Total	Average Size [bp]	Size distribution in CV [%]	Conc. [pg/µl]	Molarity [pmol/l]	Color
510	20,213	3,767.8	95	6,337	49.0	723.85	266.4	

Peak table for sample 1 : WHS1

Peak	Size [bp]	Conc. [pg/µl]	Molarity [pmol/l]	Observations
1	35	125.00	5,411.3	Lower Marker
2	5,587	281.12	76.2	
3	5,912	64.80	16.6	
4	6,237	278.89	67.8	
5	10,380	75.00	10.9	Upper Marker

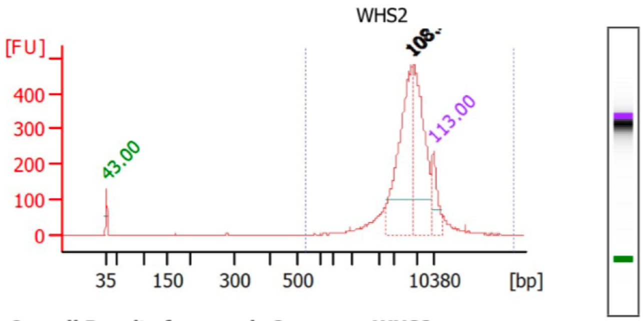

Overall Results for sample 2 : WHS2

Number of peaks found:	2
Noise:	0.9
Corr. Area 1:	3,270.3

Region table for sample 2 : WHS2

From [bp]	To [bp]	Corr. Area	% of Total	Average Size [bp]	Size distribution in CV [%]	Conc. [pg/µl]	Molarity [pmol/l]	Color
543	27,570	3,270.3	99	6,463	52.3	801.18	287.8	

Peak table for sample 2 : WHS2

Peak	Size [bp]	Conc. [pg/µl]	Molarity [pmol/l]	Observations
1	35	125.00	5,411.3	Lower Marker
2	5,875	375.19	96.8	
3	6,239	318.33	77.3	
4	10,380	75.00	10.9	Upper Marker

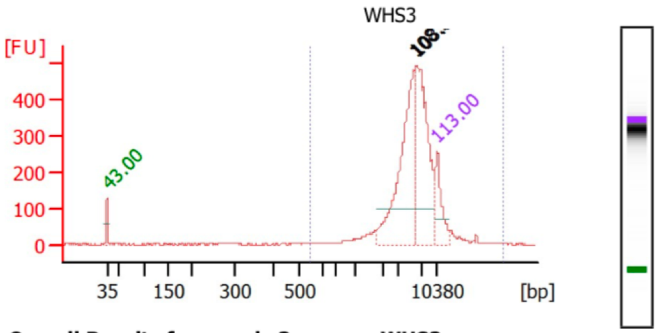

Overall Results for sample 3 : WHS3

Number of peaks found:	2
Noise:	1.0
Corr. Area 1:	3,334.6

Region table for sample 3 : WHS3

From [bp]	To [bp]	Corr. Area	% of Total	Average Size [bp]	Size distribution in CV [%]	Conc. [pg/µl]	Molarity [pmol/l]	Color
550	24,595	3,334.6	99	6,415	50.7	700.16	252.9	

Peak table for sample 3 : WHS3

Peak	Size [bp]	Conc. [pg/µl]	Molarity [pmol/l]	Observations
1	35	125.00	5,411.3	Lower Marker
2	5,903	359.39	92.2	
3	6,162	282.46	69.5	
4	10,380	75.00	10.9	Upper Marker

YNP_µT_H

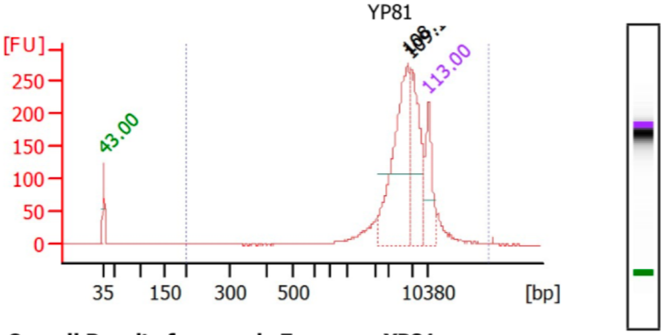

Peak table for sample 7 : YP81

Peak	Size [bp]	Conc. [pg/µl]	Molarity [pmol/l]	Observations
1	35	125.00	5,411.3	Lower Marker
2	6,023	248.28	62.5	
3	6,631	154.79	35.4	
4	10,380	75.00	10.9	Upper Marker

Overall Results for sample 7 : YP81

Number of peaks found: 2

Noise: 0.4

Corr. Area 1: 1,975.2

Region table for sample 7 : YP81

From [bp]	To [bp]	Corr. Area	% of Total	Average Size [bp]	Size distribution in CV [%]	Conc. [pg/µl]	Molarity [pmol/l]	Color
200	23,683	1,975.2	98	6,683	51.4	476.75	200.6	

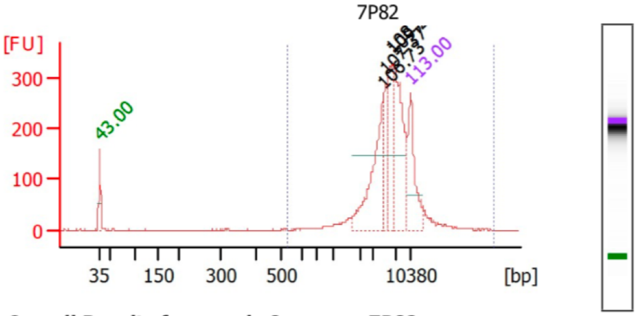

Peak table for sample 8 : 7P82

Peak	Size [bp]	Conc. [pg/µl]	Molarity [pmol/l]	Observations
1	35	125.00	5,411.3	Lower Marker
2	4,717	125.18	40.2	
3	5,213	34.76	10.1	
4	6,242	76.33	18.5	
5	6,661	136.20	31.0	
6	10,380	75.00	10.9	Upper Marker

Overall Results for sample 8 : 7P82

Number of peaks found: 4

Noise: 0.5

Corr. Area 1: 2,337.7

Region table for sample 8 : 7P82

From [bp]	To [bp]	Corr. Area	% of Total	Average Size [bp]	Size distribution in CV [%]	Conc. [pg/µl]	Molarity [pmol/l]	Color
527	29,271	2,337.7	99	6,977	54.2	413.67	147.9	

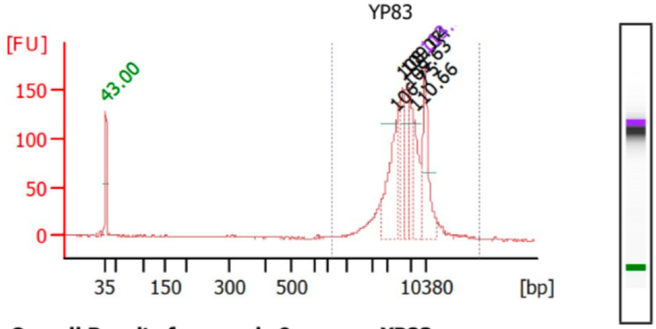

Peak table for sample 9 : YP83

Peak	Size [bp]	Conc. [pg/µl]	Molarity [pmol/l]	Observations
1	35	125.00	5,411.3	Lower Marker
2	4,728	82.02	26.3	
3	5,754	55.47	14.6	
4	6,515	43.44	10.1	
5	6,971	38.78	8.4	
6	8,002	48.61	9.2	
7	10,380	75.00	10.9	Upper Marker

Overall Results for sample 9 : YP83

Number of peaks found: 5

Noise: 0.6

Corr. Area 1: 1,046.3

Region table for sample 9 : YP83

From [bp]	To [bp]	Corr. Area	% of Total	Average Size [bp]	Size distribution in CV [%]	Conc. [pg/µl]	Molarity [pmol/l]	Color
766	22,608	1,046.3	98	6,876	46.2	332.96	107.8	

YNP_µT_Med

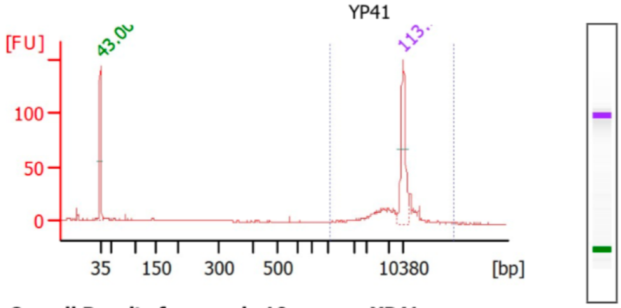

Peak table for sample 10 : YP41

Peak		Size [bp]	Conc. [pg/µl]	Molarity [pmol/l]	Observations
1	◀	35	125.00	5,411.3	Lower Marker
2	▶	10,380	75.00	10.9	Upper Marker

Overall Results for sample 10 : YP41

Number of peaks found: 0
Noise: 0.5
Corr. Area 1: 127.0

Region table for sample 10 : YP41

From [bp]	To [bp]	Corr. Area	% of Total	Average Size [bp]	Size distribution in CV [%]	Conc. [pg/µl]	Molarity [pmol/l]	Color
1,035	22,040	127.0	70	9,412	34.6	57.45	16.6	

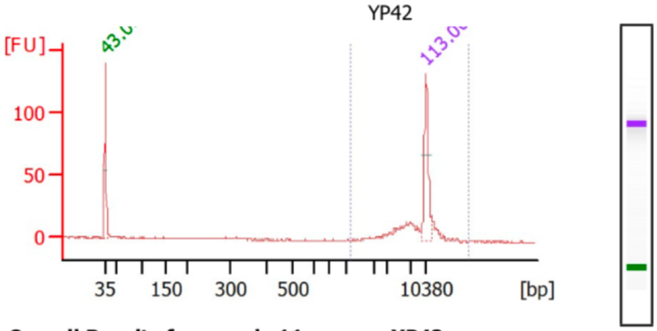

Peak table for sample 11 : YP42

Peak		Size [bp]	Conc. [pg/µl]	Molarity [pmol/l]	Observations
1	◀	35	125.00	5,411.3	Lower Marker
2	▶	10,380	75.00	10.9	Upper Marker

Overall Results for sample 11 : YP42

Number of peaks found: 0
Noise: 0.4
Corr. Area 1: 119.8

Region table for sample 11 : YP42

From [bp]	To [bp]	Corr. Area	% of Total	Average Size [bp]	Size distribution in CV [%]	Conc. [pg/µl]	Molarity [pmol/l]	Color
1,142	19,653	119.8	85	9,058	34.9	70.02	20.3	

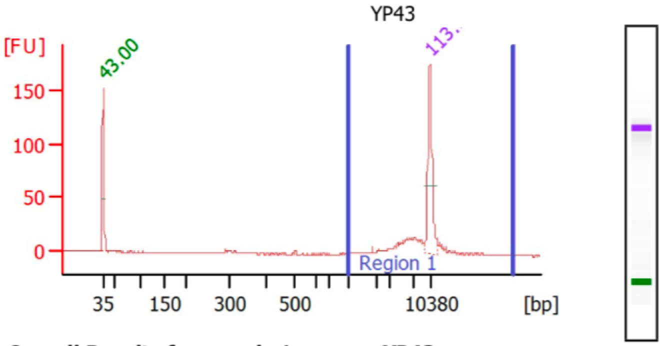

Peak table for sample 1 : YP43

Peak		Size [bp]	Conc. [pg/µl]	Molarity [pmol/l]	Observations
1	◀	35	125.00	5,411.3	Lower Marker
2	▶	10,380	75.00	10.9	Upper Marker

Overall Results for sample 1 : YP43

Number of peaks found: 0
Noise: 0.4
Corr. Area 1: 136.6

Region table for sample 1 : YP43

From [bp]	To [bp]	Corr. Area	% of Total	Average Size [bp]	Size distribution in CV [%]	Conc. [pg/µl]	Molarity [pmol/l]	Color
1,003	28,131	136.6	87	9,554	39.4	60.23	17.3	

Figure S3

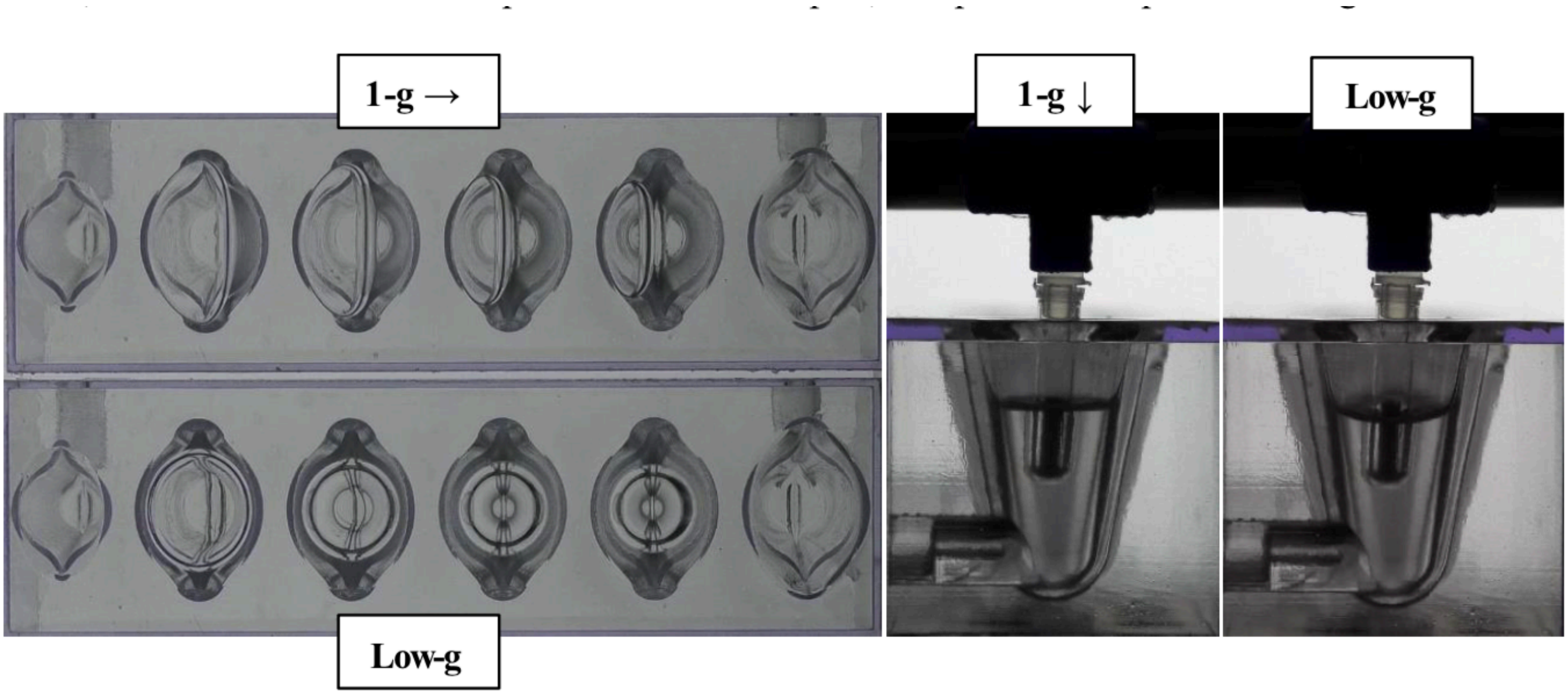

Supplement: FIGURE S1 — Summary of sequences detected by shotgun metagenomic sequencing not represented in the WCMR standard. Heatmap of counts of non-WCMR-represented species in the 18 samples and 10 controls. These non-represented species indicate contaminants from sample processing, including extraction reagents, library synthesis, or data analysis artifacts. Each sample represents the average of sample extraction triplicates. [file Image_1.PDF]
